# Control of Competing Thermodynamics and Kinetics in Vapor Phase Thin-Film Growth of Nitrides and Borides

**DOI:** 10.3389/fchem.2021.642388

**Published:** 2021-07-27

**Authors:** Isao Ohkubo, Takashi Aizawa, Katsumitsu Nakamura, Takao Mori

**Affiliations:** ^1^ International Center for Materials Nanoarchitectonics (WPI-MANA), National Institute for Materials Science (NIMS), Tsukuba, Japan; ^2^ Department of Chemistry, College of Humanities and Sciences, Nihon University, Tokyo, Japan

**Keywords:** thin-film growth, molecular beam epitaxy, chemical vapor deposition, nitrides, borides

## Abstract

Thin-film  growth is a platform technique that allows the preparation of various undeveloped materials and enables the development of novel energy generation devices. Preferred phase formation, control of crystalline orientation and quality, defect concentration, and stoichiometry in thin films are important for obtaining thin films exhibiting desired physical and chemical properties. In particular, the control of crystalline phase formation by utilizing thin-film technology favors the preparation of undeveloped materials. In this study, thin-film growth of transition metal nitride and rare-earth metal boride was performed using remote plasma–assisted molecular beam epitaxy and hybrid physical–chemical vapor deposition techniques, and was successfully achieved by tuning the competition between thermodynamics and kinetics during vapor-phase thin-film growth. Growth conditions of high crystalline quality titanium nitride thin films and high phase purity ytterbium boride thin films were not thermodynamically favorable. Appropriate control of the contribution degree of thermodynamics and kinetics during vapor-phase thin-film growth is crucial for fabricating high phase purity and high crystalline quality thin films.

## Introduction

Thin-film technology is actively used in various fundamental research, industries, and applications, and is instrumental in providing a possible solution for preparing high-quality samples of undeveloped materials such as groups of metal borides, nitrides, and carbides, and for preparing nanostructured materials (Nishinaga and Kuech, 2015). In particular, this is a fundamental technology for fabricating novel energy generation devices, including thermoelectric energy-harvesting devices. Thin-film growth under kinetically favorable growth conditions enables the formation of metastable phases, hetero-interfaces, and nanocomposite phases. In contrast, highly crystalline thin films with low defect concentrations tend to grow under thermodynamically favorable growth conditions (Dhanaraji et al., 2010; Nishinaga, 2015). Phase formation, crystalline orientation and quality, defect concentration, surface morphology, and stoichiometry in thin films are influenced by thin-film growth parameters and greatly influence their properties. Control of competing thermodynamics and kinetics during vapor-phase thin-film growth is thus necessary to obtain thin films that exhibit the required physical and chemical properties. This can be implemented by precisely tuning thin-film growth parameters. In fact, the importance of the control of competing thermodynamics and kinetics has been pointed out for thin-film growth. The in-plane and out-of-plane crystalline orientations of ZnO layers on sapphire (0001) substrates were varied by the growth temperature and the growth rate (Ohkubo et al., 1999; Ohkubo et al., 2000). The ordering degrees of Ni and Mn ions in the double perovskite La_2_NiMnO_6_ thin films were changed by the growth temperature and partial oxygen pressure. A highly ordered phase of Ni and Mn ions can be obtained at a growth temperature of approximately 700°C. Promoting the disordering of Ni and Mn ions in La_2_NiMnO_6_ thin films has been reported above 700°C (Kitamura et al., 2009).

Among the various vapor-phase thin-film growth techniques, molecular beam epitaxy (MBE) and chemical vapor deposition (CVD) are attractive because of their low-kinetic-energy growth techniques which have the potential to generate high-quality thin films with lower defect concentrations than those in films fabricated by sputtering or pulsed laser deposition techniques (Nishinaga and Kuech, 2015; Nishinaga, 2015). In addition, the control of competing thermodynamics and kinetics can be precisely realized during thin-film growth and allows the formation of the required crystalline phase in thin films. These thin-film growth techniques are used for thin-film preparation of metal nitrides, borides, and carbides for use in various energy device applications, including the preparation of thermoelectric thin films. Crystalline phase formation control using thin-film technology is effectively applied to the preparation of undeveloped materials such as nitrides, borides, and carbides. Recently, theoretical predictions by density functional theory calculations and experimental studies have suggested that layered transition metal nitrides (Ohkubo and Mori, 2014a; Ohkubo and Mori, 2014b; Ohkubo and Mori, 2015a; Ohkubo and Mori, 2015b; Orabi et al., 2015; Ohkubo and Mori, 2016), alkaline-earth metal borides (Tynell et al., 2016), and rare-earth metal borides (Guélou et al., 2018) are potential attractive thermoelectric materials.

Thin-film growth of metal nitrides, borides, and carbides composed of a transition metal and/or a rare-earth metal is associated with difficulties in terms of supplying the source material. The low vapor pressures of elemental source materials such as boron, carbon, transition metals, and rare-earth metals make it difficult to stably supply these source elements for thin-film formations, which crucially limits the growth rates and film thickness. The flux instability of these source elements is well known and generates non-stoichiometry in thin films. Few solutions exist to solve this problem. Metal–organic molecules (Ohkubo et al., 2021) and boron hydrides (Nakamura, 1984; Tynell et al., 2016; Guélou et al., 2018) are promising source materials because the vapor pressures of most of these sources are sufficiently high for thin-film preparations. The low reactivity of nitrogen gas is also a critical issue in nitride thin-film growth owing to the suppression of the formation of the nitride phase. This can be resolved by using reactive gas species of ammonia gas or nitrogen plasma sources. Control of thin-film growth can be achieved by tuning the parameters of thin-film growth temperatures and supply rates of these source materials, which are parameters related to thermodynamics and kinetics, respectively. In this study, detailed analyses of thin-film growth conditions for titanium nitride and ytterbium boride were conducted to reveal the formation of high phase purity and high crystalline quality thin films under the growth conditions of proper contribution of thermodynamics and kinetics during vapor-phase thin-film growth.

## Experimental Methods, Results, and Discussion

### Molecular Beam Epitaxy of Titanium Nitride

**FIGURE 1:**
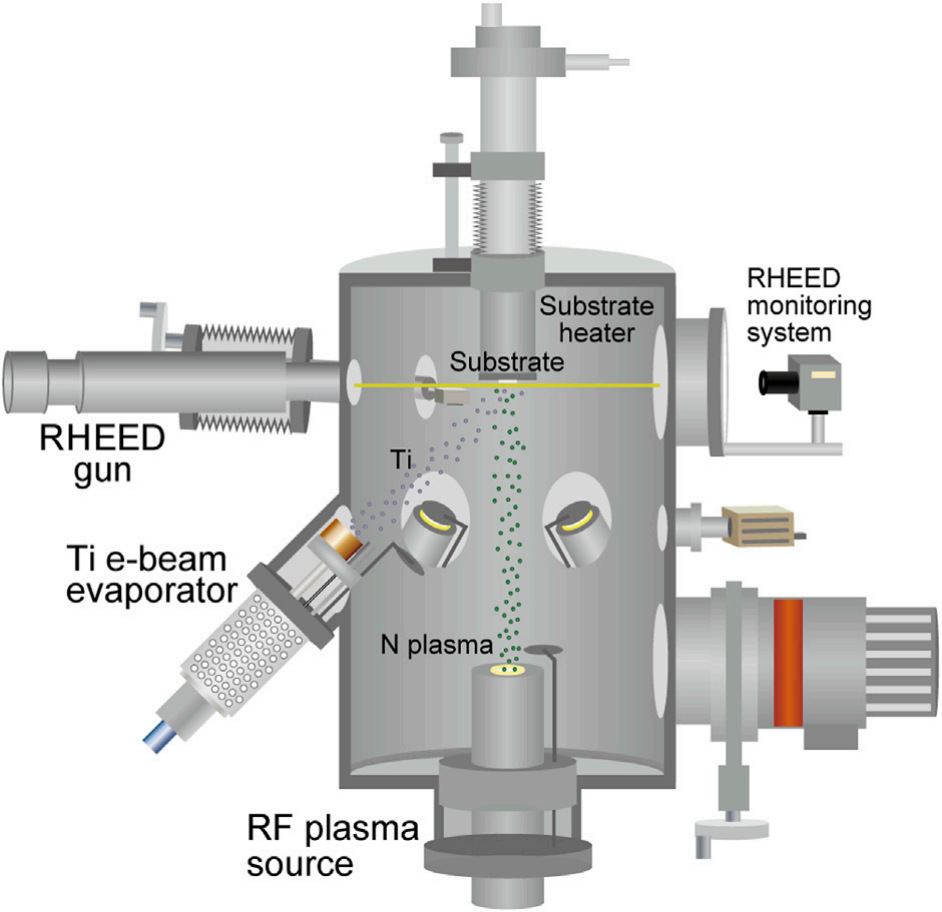
Schematic of the molecular beam epitaxy (MBE) apparatus for the thin-film growth of titanium nitrides.

**FIGURE 2:**
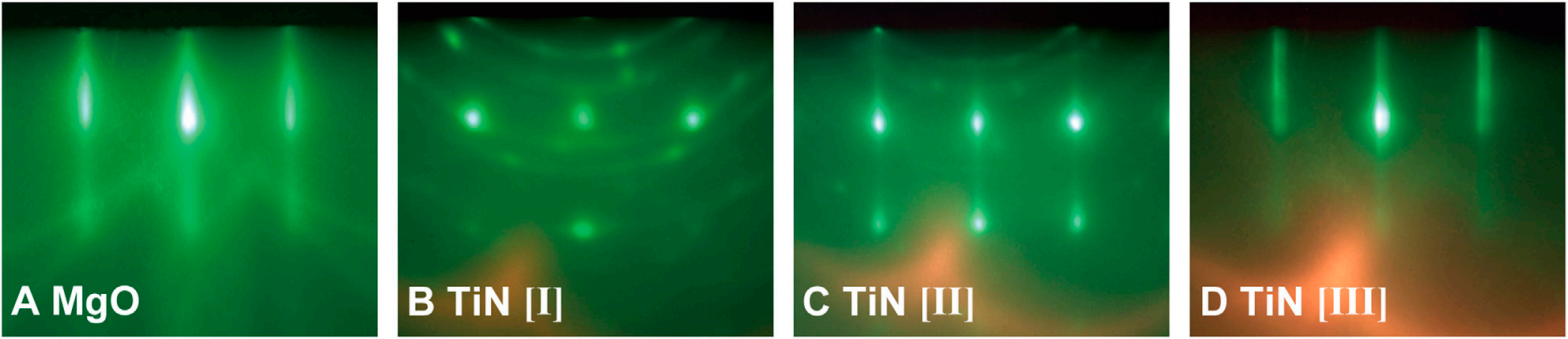
*In situ* RHEED images of (100)-oriented MgO substrates and TiN thin films along [010] azimuth. **(A)** MgO(100) substrate prior to film growth without nitrogen plasma. **(B)** TiN thin film grown at a substrate temperature of 800°C and a growth rate of 0.27 Å/s. **(C)** TiN thin film grown at 600°C and 0.92 Å/s. **(D)** TiN thin film grown at 800°C and 0.94 Å/s. All TiN layers were grown under reactive nitrogen species supply from RF plasma source operated by RF power of 300 W, 1.4 sccm N_2_ gas flow (partial nitrogen pressure of 6.0 × 10^–3^ Pa).

Among the transition metal nitrides, titanium nitride (TiN) is a remarkably important compound because of its extensive use in various different types of applications, including energy-harvesting applications (Oyama, 1996; Kafizas et al., 2013; Ohkubo et al., 2021). In this study, TiN thin films were grown on (100)-oriented single-crystal magnesium oxide (MgO) substrates (Tateho Chemical) using an MBE apparatus (Eiko), as shown schematically in Figure 1. MgO is commonly used as a lattice-matched substrate for TiN epitaxial growth because of the small lattice mismatch of 0.68% between MgO (cubic rock salt crystal structure, *a* = 4.213 Å) (Swanson and Tatge, 1953) and TiN (cubic rock salt crystal structure, *a* = 4.242 Å) (Wong-Ng et al., 1987). The base pressure of the MBE apparatus was lower than 1.0 × 10^–7^ Pa. The thin-film growth temperature (substrate temperature) was varied between 600 and 800°C. Prior to thin-film growth, preheating of the MgO(100) substrate was conducted at 800°C for 60 min under ultrahigh-vacuum conditions to obtain a clean and flat surface. As shown in Figure 2A, after preheating at 800°C for 60 min, reflection high-energy electron diffraction (RHEED) images showed a change from a broad streaky pattern to a sharp streaky pattern. Ti was supplied using an electron-beam evaporator from a Ti (99.9%, Nilaco) pellet. The supply rates of Ti were 0.2–2.3 Å/s, which were measured using a quartz crystal microbalance thickness monitor (Inficon). Reactive nitrogen species were supplied by a radio frequency (RF) remote plasma source (300 W) with a constant nitrogen gas pressure between 3.0 × 10^–3^ and 7.0 × 10^–3^ Pa (0.5–1.8 sccm N_2_ gas flow) for nitridation reactions and the formation of TiN phase on MgO (100) substrates. Film growth was monitored *in situ* using RHEED. Film thickness was evaluated using a surface profiler (Veeco, Dektak 6M). The growth rates of the TiN layers were determined from the measured film thickness, which changed with the substrate temperature, supply rate of Ti, nitrogen plasma power, and the N_2_ gas flow rate.

The RHEED patterns during TiN thin-film growth under various growth conditions are depicted in Figure 2; they varied greatly with the growth conditions. The crystalline orientation of TiN is sensitive to growth conditions. Under the condition of a growth rate lower than 0.3 Å/s, RHEED patterns exhibited Debye–Scherrer rings and weak spots, indicating that randomly oriented polycrystalline phases were dominant, even when the growth temperature was high at 800°C (Figure 2B). In contrast, dominant transmission diffraction patterns with weak rings were observed under the conditions of a growth temperature lower than 700°C and a growth rate higher than 0.3 Å/s, as shown in Figure 2C. This pattern indicates a rough surface of the grown film. Both the high growth rate (greater than 0.4 Å/s) and high growth temperature (above 700°C) were necessary to observe streaky RHEED patterns of TiN layers, which did not contain spotty and ring patterns (Figure 2D). Phase-pure epitaxial TiN growth without polycrystalline phases was achieved under the conditions of appropriately high growth rates and high growth temperature.

**FIGURE 3:**
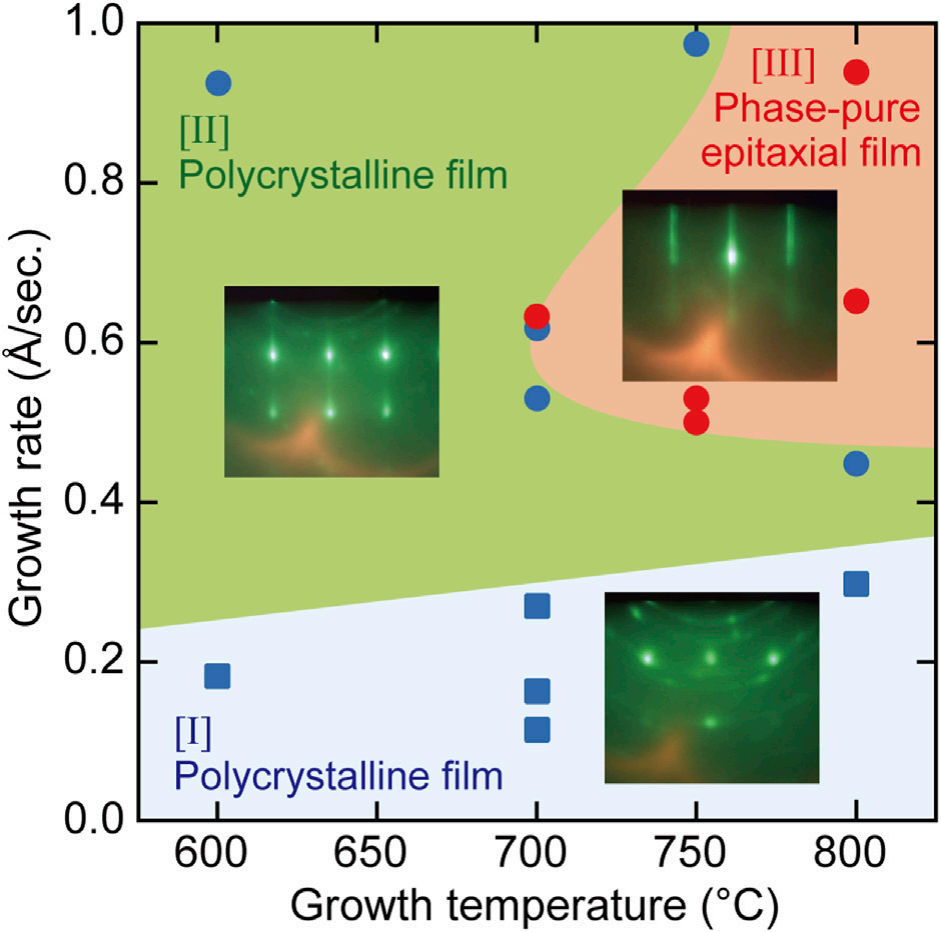
A map of TiN epitaxial growth modes depending on growth temperature and growth rate. Lines that represent borders between each region are guides for the eyes.

We mapped the growth mode of hetero-epitaxial TiN thin films on (100)-oriented MgO single-crystal substrates as a function of both the growth rate and the substrate temperature (Figure 3). The growth mode was evaluated by *in situ* RHEED, as described in Figure 2. The growth rates in Figures 2, 3 were changed by changing the supply rates of Ti and the growth temperatures under constant supply conditions of reactive nitrogen species (RF power of 300 W, 1.4 sccm N_2_ gas flow). Three distinct growth regimes of two different polycrystalline growth modes (regions [I] and [II] in Figure 3) and a phase-pure epitaxial growth mode (region [III]) were identified. Optimal growth conditions to realize phase-pure TiN epitaxial thin films with high crystalline quality and low electrical resistance were achieved at the growth conditions in region [III], where the applied growth temperature and the growth rate were 700–800°C and 0.5–1.0 Å/s, respectively. Regardless of the growth temperature, polycrystalline TiN thin films with high electrical resistance were obtained at growth rates below 0.3 Å/s (region [I]). Under the condition of low growth temperature (600–700°C) and a growth rate range of 0.2–1.0 Å/s, polycrystalline thin films with low electrical resistance were prepared (region [II]). In regions [I] and [II], streaky RHEED patterns were observed during the initial stage of TiN thin-film growth. The streaky RHEED patterns could not be maintained and were gradually changed to ring and spot patterns, as depicted in Figures 2B,C. Finally, mixed phases composed of a polycrystalline layer and an epitaxial layer were formed in regions [I] and [II]. Unsuccessful TiN homo-epitaxial growth can be considered under these growth conditions.

In general, thermodynamically favorable growth conditions are preferred to obtain a thin film with a highly aligned crystalline orientation and high crystalline quality. Low growth rate and high growth temperature are thermodynamically favorable growth conditions. However, appropriately high growth rates, greater than 0.5 Å/s, are necessary to achieve the phase-pure epitaxial TiN growth despite sufficiently high growth temperatures above 700°C (region [III]). This indicates that an appropriate epitaxial TiN growth occurs under the conditions of competing thermodynamics and kinetics. In the molecular beam epitaxy of TiN on MgO (100) substrates, the growth conditions with large thermodynamic contributions of the low growth rate and high growth temperature are located on the right-hand side of region [I] in Figure 3, where polycrystalline TiN thin films were obtained instead of phase-pure epitaxial TiN layers. In region [I], the Ti supply rate was considerably low, resulting in a large imbalance between the Ti and N supply rates. The influence of the cation/anion supply ratio on GaN MBE growth has been discussed in detail (Tarsa et al., 1997; Zywietz et al., 1998). Under nitrogen-rich growth conditions, abundantly absorbed nitrogen species on the surface tend to suppress Ga surface diffusion. Similarly, Ti surface diffusion may be suppressed by abundantly absorbed nitrogen species on the surface because of lower Ti supply rates, which could hamper the epitaxial growth of TiN even under thermodynamically favorable conditions in region [I]. The formation of phase-pure epitaxial TiN layers was achieved under the growth conditions of proper contribution of thermodynamics and kinetics. Kinetically favorable growth conditions of low growth temperature and a high growth rate are located in region [II] in Figure 3, in which polycrystalline TiN thin films were obtained. This is consistent with the crystal growth theory (Dhanaraji, G. et al., 2010; Nishinaga, 2015).

### Ytterbium Boride Thin-Film Growth *Via* Hybrid Physical–Chemical Vapor Deposition

**FIGURE 4:**
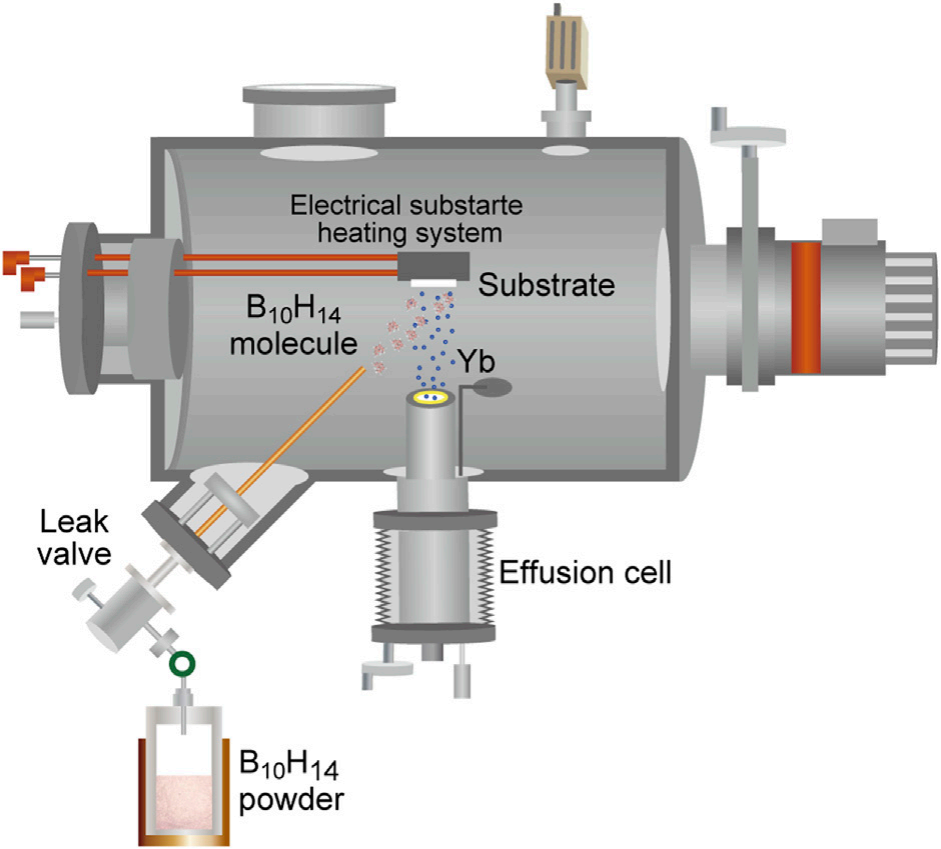
Schematic of hybrid physical–chemical vapor deposition (HPCVD) apparatus for ytterbium boride thin-film growth.

**FIGURE 5:**
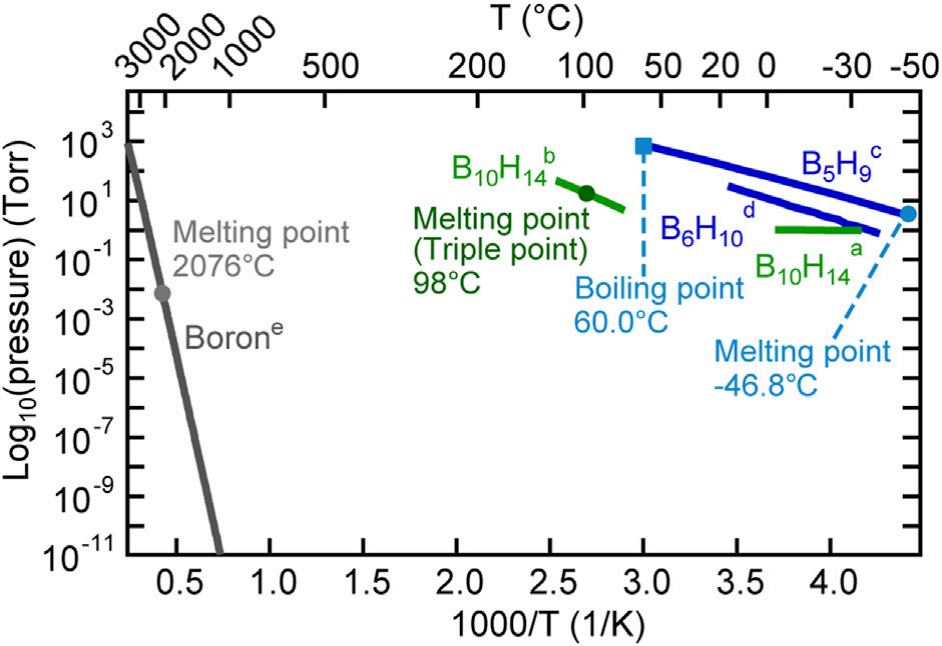
Vapor pressures of elemental boron and boron hydrides. ^a, b^ The vapor pressure data of B_10_H_14_ are taken from the literature reported by Furukawa and Park (1955) and Miller (1963). ^c^ The vapor pressure data of B_5_H_9_ are taken from the literature reported by Shapiro and Ditter (1957). ^d^ The vapor pressure data of B_6_H_10_ are taken from the literature reported by Gibbins and Shapiro (1959). ^e^ The vapor pressure data of boron are taken from the literature reported by Honig (1957).

The thermoelectric properties of borides have attracted considerable attention in recent years (Aselage and Emin, 2003; Kim and Kimura, 2011; Gürsoy et al., 2015; Slack and Morgan, 2014; Mori, 2019; Mori, 2020). Recently, relatively good thermoelectric properties of YbB_*x*_ thin films have been reported (Guélou et al., 2018). In addition, YbB_6_ has been disputed as a topological insulator that does not include the Kondo effect (Neupane et al., 2015; Kang et al., 2016; Gabani et al., 2020). Hybrid physical–chemical vapor deposition (HPCVD) was developed for the thin-film preparation of metal borides, as illustrated in Figure 4 (Tynell et al., 2016). To prepare ytterbium boride thin films, a boron hydride, decaborane (B_10_H_14_), was used as the boron source. A stable supply of boron elements from a solid boron source is rather difficult to be obtained because boron has a high melting point (2076°C) and low vapor pressure (Figure 5), and the boron melt is so reactive that it is difficult to hold it in any kind of crucible in an evaporator. The instability of supply source elements generates non-stoichiometry in thin films. On the contrary, as exhibited in Figure 5, boron hydrides are promising compounds as boron source materials for thin-film preparations owing to their high vapor pressure. A sufficiently high vapor pressure makes it possible to stably supply a boron source. However, lower boron hydrides, such as diborane (B_2_H_6_) and pentaborane (B_5_H_9_), are difficult to handle with because of their high reactivity, flammability, and toxicity. Among the boron hydrides, decaborane, which is a solid at room temperature with an adequate vapor pressure, is an appropriate compound for safe use as a source for thin-film preparations. Decaborane molecules (98%, Alfa Aesar) were supplied from a bottle and a gas line heated at approximately 390 K. Decaborane supply pressures were 1–3 × 10^−3^ Pa. Thermal sublimation of ytterbium is reasonably controllable because of its adequate vapor pressure (Daane and Habermann, 1964; Desideri et al., 1973); this was performed using a conventional effusion cell from an ytterbium ingot (99.9%, Sigma-Aldrich). Ytterbium boride thin films were prepared on (111)- and (100)-oriented MgO single-crystal substrates (Tateho Chemical) at a growth temperature (substrate temperature) range between 800 and 1,050°C, as listed in Table 1, for 30 min. Preheating of the MgO substrate was carried out at 1,000°C for 60 min prior to the thin-film growth. The substrate was heated using an electrical heating system, and the achievable temperature of the substrate was above 1,000°C. The ytterbium boride thin films were evaluated by X-ray diffraction (XRD, Rigaku SmartLab 3) and *ex situ* RHEED. The film thicknesses were characterized using a surface profiler (Veeco, Dektak 6M).

**TABLE 1:** Growth conditions of ytterbium boride thin films on MgO substrates.

Sample ID	Substrate	Growth temperature (°C)	Yb effusion cell temperature (°C)	Growth rate (Å/sec)
a	MgO (111)	800	550	17.4
b	MgO (111)	900	550	14.6
c	MgO (111)	1,000	550	9.4
d	MgO (100)	1,050	450	-

**FIGURE 6:**
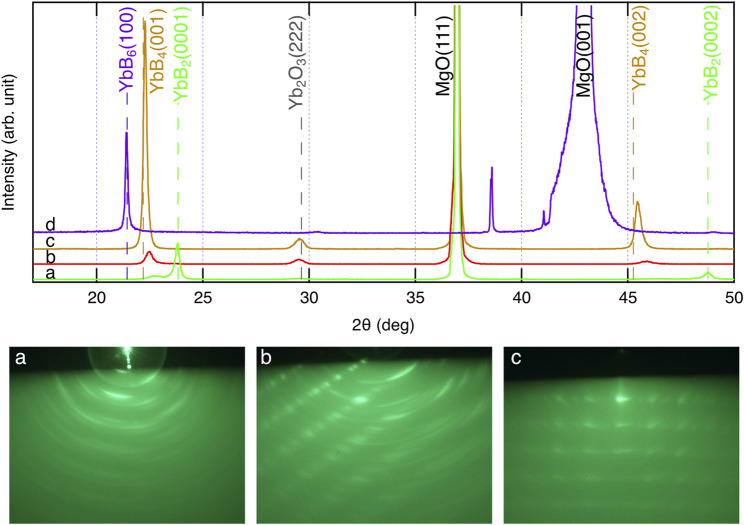
XRD **(upper panel)** and RHEED **(lower panels)** patterns of ytterbium boride thin films. The alphabets (a–d) in XRD and RHEED patterns correspond to the sample IDs listed in Table 1.

Figure 6 shows the XRD and *ex situ* RHEED patterns of the grown films. Several compounds are known to exist between Yb and B: YbB_*x*_, *x* = 2, 4, 6, 12, and 66 (Massalski, 1986). In the grown films, YbB_2_ (sample **a**), YbB_4_ (samples **b** and **c**), and YbB_6_ (sample **d**) were identified from the characteristic XRD peak from 2θ = 20°–25°. As shown in the RHEED patterns, the grown films were polycrystalline with preferred orientations. However, the preferred orientation was not necessarily perpendicular to the film surface. Therefore, the peak intensities of the XRD patterns are not proportional to the film thickness. The YbB_6_ film grown on the MgO (100) substrate (sample **d**) spontaneously peeled off after cooling. The adhesion between ytterbium boride and MgO (100) is probably very weak. This is the reason why the RHEED pattern of sample **d** could not be obtained. When the growth temperature was increased, the boron content increased, and the Yb content decreased. The Yb vapor pressure in this temperature range is significantly high, 10^3^–10^4^ Pa (Daane and Habermann, 1964; Desideri et al., 1973); thus, Yb cannot stay on the growing surface for a long time as a metal and can be easily reevaporated. Indeed, the growth rate decreased with increasing growth temperature, as shown in Table 1, probably due to the enhancement of Yb reevaporation. Consequently, boron-rich ytterbium boride became the dominant phase with increasing growth temperature. The YbB_2_ phase was grown at 800°C (sample **a**). Above 900°C, YbB_4_ was detected by XRD measurements as the dominant phase in the thin films (samples **b** and **c**). The Yb chemical potential during the film growth determines the kinetics of the YbB_*x*_ film growth. In fact, the YbB_6_ phase was grown instead of YbB_4_ under the conditions of low Yb supply rates (sample **d**). However, we have not yet obtained the YbB_12_ or YbB_66_ phases. It is likely that more thermodynamically favorable conditions are required to form complicated crystals of extremely boron-rich phases (Veremchuk et al., 2005; Sologub et al., 2017). The obtained growth rates of ytterbium boride thin films are obviously high: an order of magnitude higher than the typical thin-film growth rates of other inorganic materials. Even at high growth temperatures of 800–1,000°C, more kinetic growth conditions of a high growth rate are preferable to form the crystalline phases of ytterbium borides, probably because they lead to a reduction in the reevaporation effects of Yb at the thin film or substrate surfaces. Epitaxial stabilization effects can be expected under growth conditions with appropriate kinetic contribution (Gorbenko et al., 2002). The YbB_2_ crystal has a rather simple AlB_2_-type crystal structure (Veremchuk et al., 2005). In contrast, the crystal structures of YbB_4_ and YbB_6_ are more complicated and contain boron networks (Etourneau et al., 1979; Blomberg et al., 1995). Appropriate kinetic contributions might be necessary to crystallize YbB_4_ and YbB_6_ with complex crystal structures. A high growth rate has also been reported for the thin-film growth of SrB_6_ (Tynell et al., 2016). Appropriate control of competing thermodynamics and kinetics is necessary to obtain the crystalline phases of ytterbium borides, and this was realized by the growth conditions of a high growth rate and high growth temperature. The high growth rate of ytterbium borides was enabled by the use of boron hydride, decaborane, as a boron source, which has a sufficiently high vapor pressure. The supply of boron from a solid boron source crucially limits the achievement of a sufficient supply rate owing to its low vapor pressure. Recently, metal–organic molecular beam epitaxy of TiN has been reported (Ohkubo et al., 2021). The metal–organic source tetrakis dimethylamido titanium {TDMAT, Ti [N(CH_3_)_2_]_4_} was successfully used as a titanium source. The vapor pressure of TDMAT is also high enough for a stable supply of titanium. The use of metal–organic and boron hydride sources with sufficiently high vapor pressures can achieve stable source supplies and make it possible to control the competing thermodynamics and kinetics in vapor-phase thin-film growth.

## Conclusion

Details of titanium nitride and ytterbium boride thin-film growth *via* MBE and HPCVD methods are described. High crystalline quality epitaxial thin films of titanium nitride and high phase-pure ytterbium boride thin films were grown under the conditions of high growth temperatures and high growth rates. These growth conditions were not thermodynamically favorable conditions, even though the MBE and HPCVD methods are low kinetic energy growth techniques. The formation of high phase purity and high crystalline quality thin films were achieved under the growth conditions of proper contribution of thermodynamics and kinetics during vapor-phase thin-film growth. In many cases, phase-pure and high crystalline quality thin films are preferred to realize required physical and chemical properties. In particular, thermoelectric transport coefficients such as electrical conductivity (*σ*), Seebeck coefficient (*S*), and the thermoelectric power factor (*S* ^2^ *σ*) are greatly influenced by the crystalline phase purity, crystalline orientation, and quality. By tuning the growth parameters precisely, appropriate control of the contribution degree of thermodynamics and kinetics is crucial for fabricating thin films that exhibit the required properties.

## Data Availability

The original contributions presented in the study are included in the article/Supplementary Material; further inquiries can be directed to the corresponding author.
